# Simultaneous Detection of Adenosine Triphosphate and Glucose Based on the Cu-Fenton Reaction

**DOI:** 10.3390/s18072151

**Published:** 2018-07-04

**Authors:** Fei Qu, Jingwen Li, Wenli Han, Lian Xia, Jinmao You

**Affiliations:** 1 The Key Laboratory of Life-Organic Analysis, Qufu Normal University, Qufu 273165, China; jingwen0121@163.com (J.L.); xialian01@163.com (L.X.); youjinmao63@163.com (J.Y.); 2 Key Laboratory of Pharmaceutical Intermediates and Analysis of Natural Medicine, Qufu Normal University, Qufu 273165, China; 3 Laboratory Animal Center, Chongqing Medical University, Chongqing 400016, China; sophie0904@cqmu.edu.cn; 4 Northwest Institute of Plateau Biology, Chinese Academy of Sciences, Xining 810001, China

**Keywords:** simultaneous detection, Cu-Fenton reaction, carbon nanodots, adenosine triphosphate, glucose, satisfactory recoveries

## Abstract

Both adenosine triphosphate (ATP) and glucose are important to human health, and their abnormal levels are closely related to angiocardiopathy and hypoglycaemia. Therefore, the simultaneous determination of ATP and glucose with a single test mode is highly desirable for disease diagnostics and early recognition. Herein, a new fluorescence on/off switch sensing platform is developed by carbon nanodots (CNDs) to detect ATP and glucose simultaneously. The fluorescence of CNDs can be quenched by Cu^2+^ and hydrogen peroxide (H_2_O_2_), due to the formation of hydroxyl radicals (·OH) produced in the Cu-Fenton reaction. Based on the high affinity of Cu^2+^ with ATP, the fluorescence of CNDs will recover effectively after adding ATP. Additionally, glucose can be efficiently catalyzed by glucose oxidase (GOx) to generate H_2_O_2_, so the platform can also be utilized to analyze glucose. Under optimum conditions, this sensing platform displays excellent sensitivity and the linear ranges are from 0.1 to 7 μM for ATP with a limit of detection (LOD) of 30.2 nM, and from 0.1 to 7 mM for glucose with a LOD 39.8 μM, respectively. Benefiting from the high sensitivity and selectivity, this sensing platform is successfully applied for simultaneous detection of ATP and glucose in human serum samples with satisfactory recoveries.

## 1. Introduction

As is well known, both adenosine triphosphate (ATP) and glucose are sources of energy and important for energy metabolism. ATP supplies energy for metabolic processes and glucose is a metabolic intermediate in biological systems. Furthermore, the ATP and glucose levels in blood are indicators of human health conditions. For example, the excessive production of ATP by creatine kinase is the pathogenesis of angiocardiopathy [1] and the aberrant concentration of ATP will cause energy disturbance, resulting in hypoglycaemia, ischemia, and Parkinsons’ disease [2]. On the other hand, the high glucose levels will produce diabetes mellitus, which damages the eyes, kidneys, feet, and heart, and also causes a series of angiocardiopathy [3], while hypoglycaemia is usually due to the low concentration of glucose. This means that both the levels of ATP and glucose are related to angiocardiopathy and hypoglycaemia by virtue of the coexistence of ATP and glucose in biological samples [4,5,6,7], thus, the simultaneous determination of ATP and glucose with a single test mode is highly desirable for disease diagnostics and early recognition.

A comparison with the traditional methods, such as chromatography and electrochemical techniques [8,9], fluorescence analysis receives a great deal of attention due to its simplicity, rapid response, and high selectivity. The fluorescence sensing strategy for ATP is usually developed according to the reaction between ATP and aptamer or between ATP and copper ions (Cu^2+^). For example, ATP-aptamer complexes provided greater protection for gold nanoparticles (AuNPs) against salt-induced aggregation than either aptamer or ATP alone, and the dispersive AuNPs rather than aggregation could efficiently quench the fluorescence of Tb ion-functionalized carbon dots due to the fluorescence resonance energy transfer [10]. In addition, compared with other cations, ATP has a strong affinity for Cu^2+^, which can completely quench the emission of perylene diimide functionalized with histidine. Thus, the fluorescence recovery positively correlated with ATP concentrations [11]. On the other hand, many fluorescence glucose sensors based on new fluorescence transduction schemes are already developed [12]. In these reports, the enzyme glucose oxidase (GOx) has been widely employed in glucose sensing because hydrogen peroxide (H_2_O_2_) is the main product of the reaction between glucose and GOx. For instance, H_2_O_2_ formed in the oxidization of glucose by GOx, resulting in the fluorescence quenching of carbon nanodots (CNDs) in the presence of Fe^2+^, and the concentration of glucose could be measured indirectly [13]. Although these reports showed good sensitivity and selectivity, these strategies could not incorporate both elements which responded, respectively, to Cu^2+^ and H_2_O_2_, so the simultaneous measurement of ATP and glucose could not be achieved. Up to now, only an amperometric biosensor was reported for the simultaneous determination of ATP and glucose [14], and other ways, especially fluorescence techniques, have not been presented.

Herein, it is found that the complex of Cu^2+^ and H_2_O_2_, rather than either Cu^2+^ or H_2_O_2_ alone, can quench the fluorescence of CNDs due to the hydroxyl radicals (·OH) produced in the Cu-Fenton reaction [15,16,17]. By virtue of the binding of ATP to Cu^2+^ and arising H_2_O_2_ in the reaction between glucose and GOx, ATP and glucose can be simultaneously detected. The linear range of ATP is 0.1 to 7 µM and the linear response of glucose is from 0.1 to 7 mM. Benefiting from the superior sensitivity and selectivity, this sensing platform is successfully applied for simultaneous detection of ATP and glucose in human serum samples with satisfactory recoveries.

## 2. Materials and Methods

### 2.1. Chemicals and Materials

Ascorbic acid (AA), hydrogen peroxide (H_2_O_2_), glucose oxidase (GOx), glucose, galactose, fructose, sucrose, mannose, lactose, sodium citrate, adenosine triphosphate (ATP), cytidine 5′-triphosphate disodium salt (CTP), adenosine 5′-diphosphate (ADP), uridine-5′-triphosphate (UTP), guanosine triphosphate (GTP) were purchased from Aladdin (Shanghai, China). CH_3_COONa, HCl, KCl and CuSO_4_ were obtained from Shanghai Shenbo Chemical Co., Ltd., Shanghai, China. All reagents were of analytical grade. All the solutions were prepared using ultrapure water produced with a Millipore-Q water system.

### 2.2. Instruments

The fluorescence spectra were recorded with a Hitachi F-7000 fluorescence spectrophotometer (Tokyo, Japan). Transmission electron microscopy (TEM) images were recorded on a JEM-2100PLUS (JEOL, Japan). The Fourier transform infrared (FT-IR) spectra of the samples were analyzed using Thermo Nicolet Nexus 470 FT-IR ESP spectrometer (Nicolet, WI, USA). The ultraviolet-visible (UV-vis) absorption spectra were obtained on a Cary 300 Bio UV-vis spectrophotometer (Varian, Palo Alto, CA, USA) and the pH values of solutions were measured using a pH meter (Mettler Toledo FE20, Zurich, Switzerland).

### 2.3. Synthesis of CNDs

Typically, 0.8 g AA was dissolved in 20 mL water. Then the above solution was stirred thoroughly and then transferred into a 50 mL autoclave reactor. The reactor was heated to 160 °C in a constant temperature drying oven for 70 min. After cooling to room temperature, a clear yellow aqueous dispersion containing CNDs was gained. Subsequently, the resulting yellow solution (20 mL) was dried in a drying oven at 60 °C to constant weight (for about 14 h). Then, 39 mg powder of carbon nanodots was weighed and diluted in 1 mL water to obtain CNDs solution (39 mg/mL). The prepared solution of CNDs was diluted 100 times before use, and the concentration was 0.39 mg/mL.

### 2.4. Fluorescence Responses of Cu^2+^

The fluorescence detection of Cu^2+^ was performed as follows: briefly, 35 µL CNDs (0.39 mg/mL), 50 µL H_2_O_2_ (0.1 M), different concentrations of Cu^2+^, 200 µL HCl-CH_3_COONa buffers (pH = 5) and water were mixed together, and the final volume was 1 mL. After incubation for 10 min at room temperature, the fluorescence of the mixture was measured at 380 nm with an excitation of 315 nm. With increasing the concentration of Cu^2+^, the fluorescence of CNDs decreased linearly and the fluorescence differences were expressed as F_0_-F, in which F_0_ and F represented the fluorescence intensities of CNDs-H_2_O_2_ in the absence and presence of Cu^2+^, respectively.

Additionally, the LOD is the limit of detection, which is defined as the concentration of analyte that corresponds to three times the signal-to-noise ratio (S/N = 3) [18]. LOD is calculated according to the expression LOD = 3σ/K, where σ is the standard deviation for the blank solution (*n* = 10), and K is the slope of the calibration curve.

### 2.5. Fluorescent H_2_O_2_ Assay

A typical H_2_O_2_ detection procedure was conducted as follows: Firstly, 35 µL CNDs (0.39 mg/mL), 60 µL Cu^2+^ (0.01 mM), 200 µL HCl-CH_3_COONa buffers (pH = 5), various concentrations of H_2_O_2_ and water were mixed thoroughly. The final volume of the mixture was 1 mL. After incubation for 10 min at ambient temperature, the concentration of H_2_O_2_ linearly responded to the fluorescence differences of F_1_-F_2_ (F_1_ and F_2_ represented the fluorescence intensity of CNDs-Cu^2+^ in the absence and presence of H_2_O_2_).

### 2.6. Fluorescence Detection of ATP

The assay was carried out as follows: (i) 200 µL HCl-CH_3_COONa buffers (pH = 5), 60 µL Cu^2+^ (0.01 mM) and ATP with different concentrations were added to a certain amount of water and reacted for 10 min at room temperature; (ii) 35 µL CNDs (0.39 mg/mL) and 50 µL H_2_O_2_ (0.1 M) were injected to the mixture and the volume of final solution was 1 mL. After the mixture reacted for 10 min at room temperature, a linear correlation was found between the concentration of ATP and the fluorescence differences of F_4_ − F_3_, in which F_3_ was the fluorescence intensity of CNDs-Cu^2+^-H_2_O_2_ system and F_4_ was the fluorescence intensity of the system with the addition of various concentrations of ATP.

### 2.7. Fluorescence Measurement of Glucose

Firstly, the mixture containing 13 μL GOx (765 U/mL) and different concentrations of glucose was incubated at 37 °C for 60 min, and then 200 µL HCl-CH_3_COONa buffers (pH = 5), 60 μL Cu^2+^ (0.01 mM) and 35 µL CNDs (0.39 mg/mL) were added to the mixture. Ten minutes later, their fluorescence spectra were recorded. The concentration of glucose linearly responded to the fluorescence differences of F_5_ − F_6_, where F_5_ and F_6_ were the fluorescence of CNDs-Cu^2+^-GOx system in the absence and presence of glucose.

### 2.8. Real Sample Detection

The real sample detection in our assay was carried out as follows: Typically, a 1.5 mL blood sample was kept at 37 °C in a water bath for about 0.5 h. Then, 3.0 mL acetonitrile was added and followed by centrifugation at 4000 rpm for 20 min to remove large molecules and proteins. Subsequently, the supernatant was removed and kept in a water bath (60 °C) for about 1 h to evaporate acetonitrile. The supernatant of the serum sample was diluted 10 times and measured by the present sensing system to detect ATP and glucose. Then, the standard ATP and glucose solutions with different concentrations were spiked into the human serum samples, respectively, to investigate the reliability of this assay.

### 2.9. Validation Test of TMB

The validation of the Cu-Fenton activity was performed as follows: the experiments were performed in 200 µL HCl-CH_3_COONa buffers (pH = 5) containing 5 mM TMB, 500 mM H_2_O_2_ and differing concentrations of Cu^2+^ followed by the addition of water to a final volume of 1 mL. After reaction for 10 min, the colour was present in these samples.

## 3. Results and Discussion

### 3.1. Characterization of CNDs

**Figure 1:**
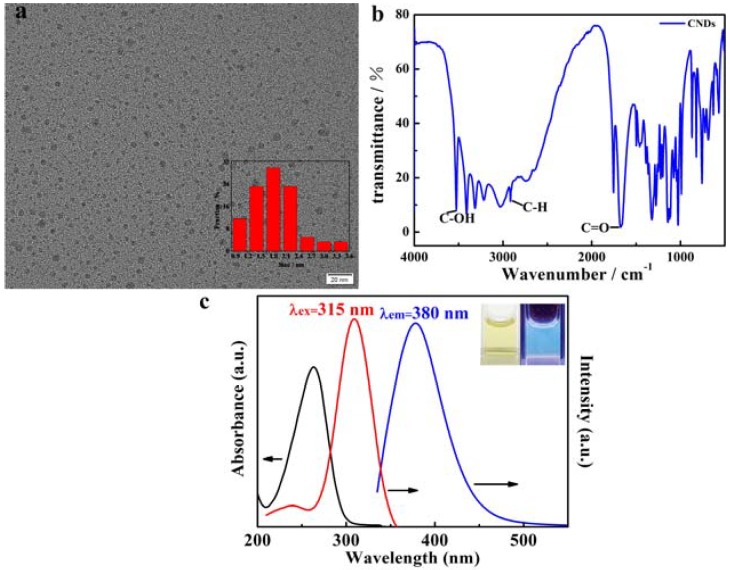
TEM image (**a**), FT-IR spectrum (**b**), UV–VIS absorption (black curve), and fluorescence emission (blue curve), and excitation (red curve) spectra of CNDs (**c**). The inset of (**c**) is the photograph of CNDs under visible light and UV light at 365 nm.

As shown in the TEM image (Figure 1a), the CNDs exhibit an approximately spherical shape and are well-dispersed. These nanoparticles have a size distribution from 1 to 3.4 nm and the average diameter of CNDs is about 1.8 nm by estimating the size of 100 dots in the TEM image. The structure and composition of CNDs are characterized by FT-IR spectroscopy (Figure 1b). The CNDs have a main absorption of C-H stretching vibration at 2900 cm^−1^, the C=O stretching at 1740 cm^−1^, and the peak at 3452 cm^−1^ is related to the C-OH bond stretching vibrations. It demonstrates the presence of oxygen-containing functional groups such as hydroxyl and carbonyl groups on the surface of particles. Additionally, these nanodots show a strong peak at 260 nm (Figure 1c), which is ascribed to π-π* transition of carbon [19]. Next, the emission is found to be excitation-dependent, including wavelength and intensity in a well-regulated mode within the exciting range of 285–345 nm (Figure S1). Although the emission peak shifts from 375 to 390 nm with increasing excitation wavelengths, its intensity reaches a maximum at 315 nm excitation (Figure 1c). The excitation-dependent emission spectra have been reported by various works involving CNDs [20,21]. Meanwhile, the CNDs solution under UV light exhibits bright blue fluorescence, whereas it is yellow under daylight.

### 3.2. Fluorescence Quenching of CNDs

**Figure 2:**
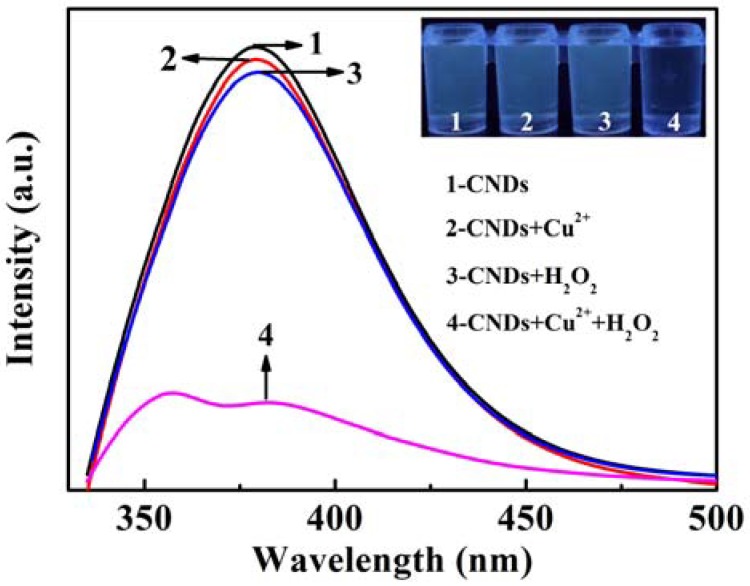
Fluorescence spectra of CNDs in the absence and presence of Cu^2+^, H_2_O_2_, and the mixture of Cu^2+^ and H_2_O_2_, respectively. The concentrations of CNDs, Cu^2+^, and H_2_O_2_ were 0.014 mg/mL, 2 µM, and 5 mM, respectively.

**Scheme 1:**
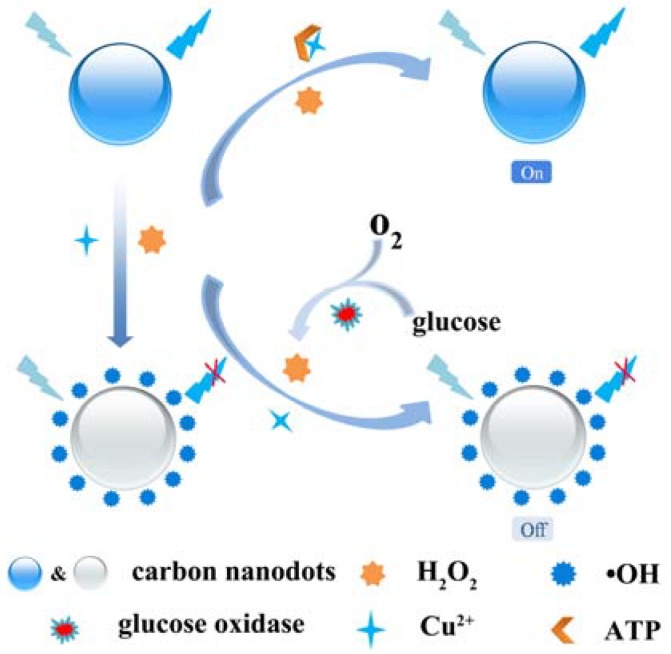
Schematic illustration of the CNDs as a fluorescence probe for the detection of ATP and glucose.

According to Figure 2, when only H_2_O_2_ or Cu^2+^ is mixed with CNDs, the emission at 380 nm changes inconspicuously. However, by both adding H_2_O_2_ and Cu^2+^, the fluorescence of CNDs is quenched effectively. While the amount of H_2_O_2_ is fixed, the emission of CNDs sensitively responds to the concentration of Cu^2+^ in the range from 1 nM to 2 μM with LOD 0.65 nM (Figure S2), which is lower than most works [22,23]. Additionally, it should be noted that when the concentration of Cu^2+^ is higher than 4 μM (Figure S3), it can directly quench the fluorescence of CNDs without the addition of H_2_O_2_, which is consistent with previous literature [24], but Cu^2+^ ions with a low amount (especially below 2 μM) is invalid. Additionally, with the unchanged amount of Cu^2+^, the fluorescence of these nanoparticles also decreases linearly when the concentration of H_2_O_2_ increases from 25 µM to 5 mM with LOD 7.8 µM (Figure S4). Like the Fenton reaction between Fe^2+^ and H_2_O_2_, Cu^2+^ and H_2_O_2_ can also produce the reactive ·OH. In this process, Cu^2+^ oxidizes H_2_O_2_ to generate O_2_^•-^and Cu^+^, and Cu^+^ is able to react with excess H_2_O_2_ to form ·OH [25,26,27,28], which can catalyse the oxidation of TMB to produce the blue colour reaction (Figure S5). TMB oxidation-induced colour development is positively correlated with Cu^2+^ concentrations. Therefore, the ·OH is believed to quench the fluorescence of CNDs, and the quenching efficiency reaches the maximum at pH 5 (Figure S6). Cu-Fenton reaction is different from the Fe-Fenton reaction, and it usually requires a small amount of Cu^2+^ and a large amount of H_2_O_2_, which just correspond to the low concentration of ATP and high concentration of glucose in human serum. Based on the binding of ATP to Cu^2+^ and arising H_2_O_2_ in the reaction between glucose and GOx, ATP, and glucose can be simultaneously detected with a single test mode (Scheme 1).

### 3.3. Fluorescence Recovery of CNDs-Cu^2+^-H_2_O_2_ System by ATP

**Figure 3:**
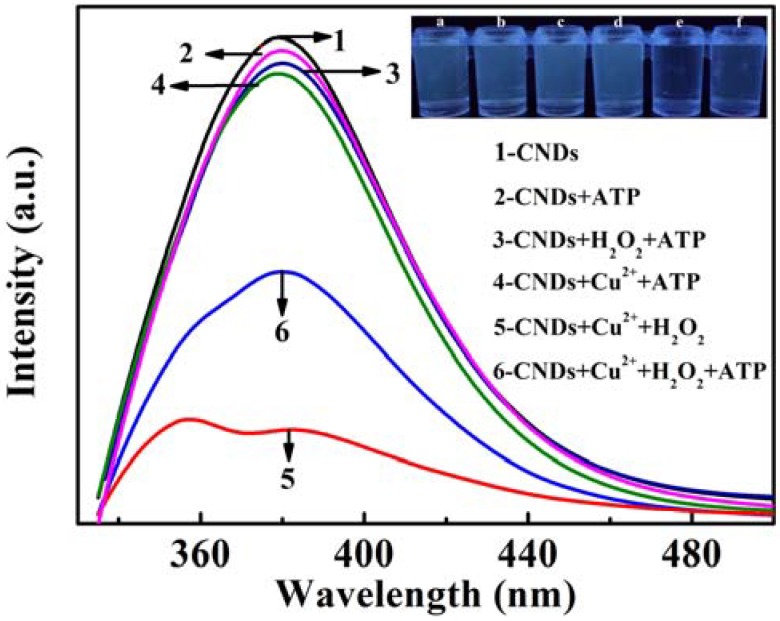
Fluorescence spectra of free CNDs (**1**) and CNDs in the presence of ATP (**2**), H_2_O_2_ and ATP (**3**), Cu^2+^ and ATP (**4**), Cu^2+^ and H_2_O_2_ (**5**) and Cu^2+^, H_2_O_2_, and ATP (**6**). The final concentrations of CNDs, H_2_O_2_, Cu^2+^, and ATP were 0.014 mg/mL, 5 mM, 600 nM and 7 µM respectively. The inset shows the corresponding images under UV light.

**Figure 4:**
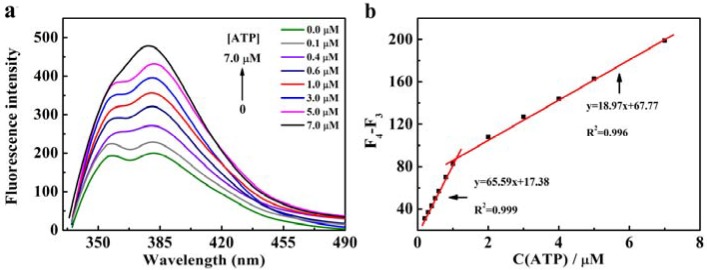
Fluorescence responses to different concentrations of ATP (**a**) and corresponding linear ranges (**b**). The concentrations of CNDs, H_2_O_2_ and Cu^2+^ were 0.014 mg/mL, 5 mM and 600 nM, respectively.

As shown in Figure 3, when the ATP is introduced in the CNDs-Cu^2+^-H_2_O_2_ system, the quenched fluorescence recovers effectively because Cu^2+^ ions exhibit a strong binding affinity to ATP with respect to multiple phosphates, while ATP alone will not influence the fluorescence of CNDs, CNDs-Cu^2+^, and CNDs-H_2_O_2_. All of results indicate that ATP indeed binds with Cu^2+^ ions, which cannot react with H_2_O_2_ to form ·OH. At the same time, the reaction between ATP and Cu^2+^ ions can complete within 10 min, suggesting a rapid reaction rate (Figure S7). The enhanced fluorescence positively correlates with the concentration of ATP in the linear range from 0.1 to 7 µM with LOD 30.2 nM (Figure 4). Comparing with the previous reports (Table 1), our method is not inferior to others [29,30,31,32,33,34,35] and also exhibits relatively wide linear ranges and a low limit of detection of ATP.

### 3.4. Detection of Glucose Based on CNDs-Cu^2+^ System

**Figure 5:**
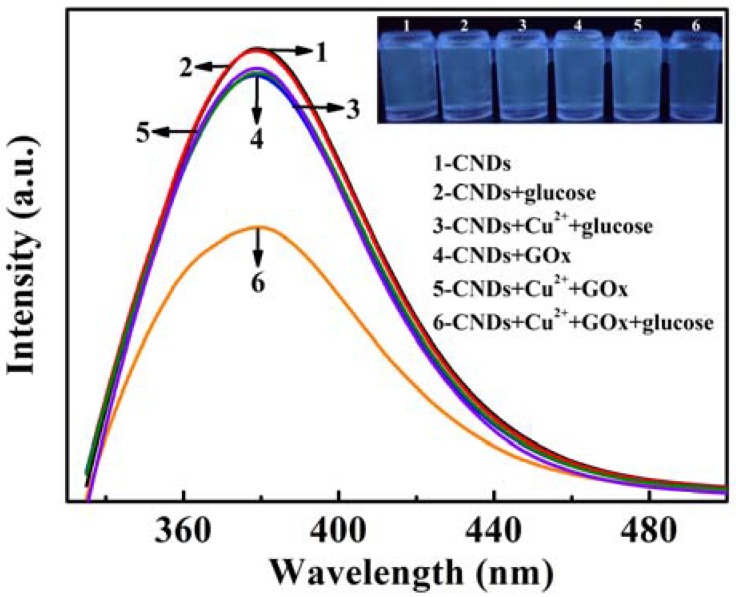
Fluorescence spectra of free CNDs (**1**) and CNDs in the presence of glucose (**2**), Cu^2+^ and glucose (**3**), GOx (**4**), Cu^2+^ and GOx (**5**), and the mixture of Cu^2+^, glucose and GOx (**6**). The final concentrations of CNDs, glucose, Cu^2+^ and GOx were 0.014 mg/mL, 5 mM, 600 nM and 10 U/mL respectively. The inset shows the corresponding images under UV light at 365 nm.

**Figure 6:**
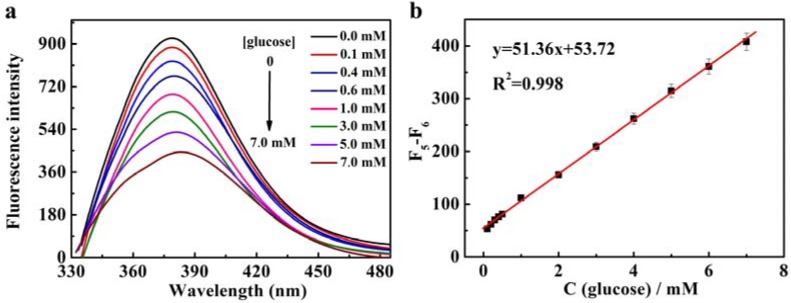
Fluorescence spectra of CNDs in the presence of different concentrations of glucose (**a**) and the corresponding linear range (**b**). The concentrations of CNDs, Cu^2+^ and GOx were 0.014 mg/mL, 600 nM, and 10 U/mL, respectively.

H_2_O_2_ is the main product of the reaction between glucose and GOx, so CNDs-Cu^2+^-H_2_O_2_ system can be utilized to analyse glucose. As illustrated in Figure 5, there is no significant difference when glucose or GOx is added in CNDs and CNDs-Cu^2+^ system. When glucose is oxidized by GOx, the fluorescence of CNDs-Cu^2+^ platform was quenched effectively. The fluorescence difference was proportional to the concentration of glucose in the range from 0.1 to 7 mM with LOD 39.8 µM (Figure 6). The fluorescence response of this platform to the glucose ranging from 0 to 1.0 mM is depicted in Figure S8, where the slope of the linear equation is similar to that of glucose with concentration from 0.1 to 7.0 mM, indicating that the glucose with low concentrations can be also sensitively detected. In comparison with the reported studies (Table 2), this new strategy is demonstrated to not be inferior to other works [36,37,38,39,40,41]. Additionally, CNDs involved in this assay, as a novel probe, are easily synthesized, experimentally convenient, and low-cost, and can be used to detect ATP and glucose simultaneously.

### 3.5. Selectivity for ATP and Glucose

**Figure 7:**
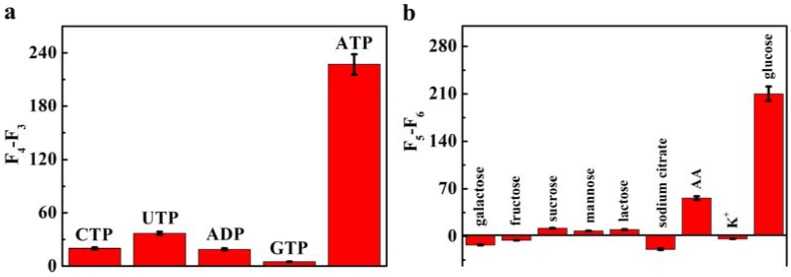
Selectivity of detection of ATP (**a**) and glucose (**b**). The concentrations of ATP and other interference substances in Figure 7a are 3 µM. In Figure 7b, glucose, and other substances are 3 mM.

To examine the selectivity of this CNDs-Cu^2+^-H_2_O_2_ system for ATP, other analogous molecules, including CTP, ADP, UTP, and GTP are investigated under the optimized conditions. As shown in Figure 7a, only the addition of ATP produces an obvious fluorescence recovery, suggesting the highly selectivity for ATP. Additionally, some interference substances, such as galactose, fructose, sucrose, mannose, lactose, sodium citrate, AA, and KCl, are evaluated. Due to the high specificity of GOx for glucose, other compounds cannot induce the effective fluorescence quenching of CNDs, indicating a high selectivity of our proposed sensing system for glucose.

### 3.6. Detection of ATP and Glucose in Human Serum Samples

To evaluate the applicability of this sensing system, the proposed strategy is used to determine ATP and glucose in human serum samples. The results are shown in Table 3, the concentration of ATP in human serum sample (without dilution) measured by our method is about 4.3 µM, which is consistent with the value detected by the high-performance liquid chromatograph (HPLC). With a standard addition method, the real samples are spiked with certain amounts of ATP and the satisfactory recoveries of ATP in the range from 102.4 to 106.4% are reached. On the other hand, the concentration of glucose in human serum sample (without dilution) detected by our method is about 3.41 mM, which is similar to the clinic value provided by local hospital (3.78 mM). Next, the recoveries in the ranges from 98.0 to 104.8% are obtained by spiking different concentrations standard glucose solutions into human serum samples (Table 4). All results demonstrate that this proposed method is practicable, reliable, and can be used for simultaneous determination of ATP and glucose in human serum samples.

## 4. Conclusions

Herein, a fluorescence sensing platform based on CNDs for the detection of ATP and glucose with superior sensitivity and selectivity has constructed. The sensing mechanism is that Cu^2+^ can react with H_2_O_2_ to generate ·OH, which quenches the fluorescence of CNDs. Incorporating both ATP and glucose, which respond respectively to Cu^2+^ and H_2_O_2_, the measurements of ATP and glucose can be simultaneously achieved in human serum samples with satisfactory recoveries. Due to the abnormal levels of ATP and glucose closely related to some diseases, this assay has been proposed in an effort to find a proper candidate for use in routine clinical practice.

## Figures and Tables

**Table 1:** Comparison of analysis methods for the detection of ATP.

Method	Probe	Linear Range	LOD	Ref.
Fluorescence	Ru complex	0–0.1 µM	20 nM	29
Fluorescence	DNA template Ag nanoclusters (AgNCs)	0.5–8.0 µM	91.6 nM	30
Colorimetric detection	Gold nanoparticles	4.4–132.7 µM	0.6 µM	31
Fluorescence	Cysteamine capped CdS quantum dots (QDs)	20–80 µM	17 µM	32
Fluorescence	DNA binding dye berberine	0.5–50 µM	140 nM	1
Fluorescence	QD-tagged aptamer	0.1–1 mM	24 µM	33
Electrochemical	labeled anti-ATP aptamer	10 nM–1 mM	-	34
Fluorescence and Colorimetric detection	cationic polythiophene derivative	10^−8^–10^−4^ M	-	35
Fluorescence	CNDs	0.1–7 µM	30.2 nM	This work

**Table 2:** Comparison of analysis methods for the detection of glucose.

Method	Probe	Linear Range	LOD	Ref.
Colorimetric detection	MoS_2_ nanosheets	5–150 µM	1.2 µM	36
Fluorescence	boronic acid modified carbon dots	9–900 µM	1.5 µM	19
Colorimetric detection	Gold nanoparticles	0–5 mM	-	37
Colorimetric detection	ZnS nanoparticles	0.05–0.5 mM	36 µM	38
Fluorescence	Copper nanoclusters	10–100 µM	8 µM	39
Colorimetric detection	Prussian blue nanoparticles	0.1–50 µM	0.03 µM	40
Fluorescence	carbon nitride dots	1–100 µM	0.4 µM	41
Fluorescence	CNDs	0.1–7 mM	39.8 µM	This work

**Table 3:** Determination of ATP in serum samples.

Sample	Standard Added µM	Found Value µM	Recovery (%)	RSD (*n* = 3, %)
1	0.0	0.433 *	-	1.43
2	0.1	0.537	104.0	1.68
3	1.0	1.497	106.4	1.16
4	5.0	5.553	102.4	1.61

* The concentration of ATP in human serum sample with diluted 10 times measured by our method is 0.43 µM, which is in agreement with the values detected by HPLC (4.1 µM without dilution).

**Table 4:** Determination of glucose in serum samples.

Sample	Standard Added mM	Found Value mM	Recovery (%)	RSD (*n* = 3, %)
1	0	0.341 *	-	1.38
2	0.1	0.439	98.0	1.02
3	1.0	1.389	104.8	1.52
4	3.0	3.284	98.1	1.83

* The concentration of glucose in human serum sample with diluted 10 times measured by our method is 0.341 mM, which is consistent with the clinical data provided by a local hospital (3.78 mM without dilution).

## Supplementary Materials

The following are available online at http://www.mdpi.com/1424-8220/18/7/2151/s1, Figure S1. Excitation-dependent fluorescence spectra of CNDs. Figure S2. Fluorescence spectra of CNDs upon addition of different concentrations of Cu^2+^ (**a**) in the presence of H_2_O_2_ (5 mM) and the corresponding linear ranges of Cu^2+^ (**b**). Figure S3. Fluorescence spectra of CNDs upon addition of different concentrations of Cu^2+^ in the absence of H_2_O_2_. Figure S4. Fluorescence spectra of CNDs upon addition of different concentrations of H_2_O_2_ (**a**) in the presence of Cu^2+^ (2 µM) and the corresponding linear ranges of H_2_O_2_ (**b**). Figure S5. Photographs of Cu^2+^-H_2_O_2_-TMB system with different concentrations of Cu^2+^ under visible light. Figure S6. Influence of pH values on CNDs-Cu^2+^-H_2_O_2_ system. Figure S7. Influence of the incubation time between Cu^2+^and ATP. The concentrations of CNDs, H_2_O_2_, Cu^2+^ and ATP were 0.014 mg/mL, 5 mM, 600 nM and 1 µM, respectively. Figure S8. Fluorescence spectra of CNDs in the presence of different concentrations of glucose (**a**) and the corresponding linear range from 0.1 mM to 1 mM (**b**). The concentrations of CNDs, Cu^2+^, and GOx were 0.014 mg/mL, 600 nM, and 10 U/mL respectively.

## References

[1] Wei Y., Chen Y., Li H., Shuang S., Dong C., Wang G. (2015). An exonuclease I-based label-free fluorometric aptasensor for adenosine triphosphate (ATP) detection with a wide concentration range. Biosens. Bioelectron..

[2] Ma K., Wang H., Li H., Wang S., Li X., Xu B., Tian W. (2016). A label-free aptasensor for turn-on fluorescent detection of ATP based on AIE-active probe and water-soluble carbon nanotubes. Sens. Actuators B.

[3] Hao G., Wang D., Sun Y., Yu J., Lin F., Cao H. (2017). Association of blood glucose and lipid levels with complete blood count indices to establish a regression model. Biomed. Rep..

[4] Zhu J., Yu C., Chen Y., Shin J., Cao Q., Kim J. (2017). A self-assembled amphiphilic imidazolium-based ATP probe. Chem. Commun..

[5] He H., Ma V.P.Y., Leung K.H., Chan D.S.H., Yang H., Cheng Z., Leung C.H., Ma D.L. (2012). A label-free G-quadruplex-based switch-on fluorescence assay for the selective detection of ATP. Analyst.

[6] Rastogi L., Karunasagar D., Sashidhar R.B., Giri A. (2017). Peroxidase-like activity of gum kondagogu reduced/stabilized palladium nanoparticles and its analytical application for colorimetric detection of glucose in biological samples. Sens. Actuators B.

[7] Hu A., Liu Y., Deng H., Hong G., Liu A.L., Lin X., Xia X., Chen W. (2014). Fluorescent hydrogen peroxide sensor based on cupric oxide nanoparticles and its application for glucose and l-lactate detection. Biosens. Bioelectron..

[8] Lu L., Si J., Gao Z., Zhang Y., Lei J., Luo H., Li N. (2015). Highly selective and sensitive electrochemical biosensor for ATP based on the dual strategy integrating the cofactor-dependent enzymatic ligation reaction with self-cleaving DNAzyme-amplified electrochemical detection. Biosens. Bioelectron..

[9] Schweinsberg P.D., Loo T.L. (1980). Simultaneous analysis of ATP, ADP, AMP, and other purines in human erythrocytes by high-performance liquid chromatography. J. Chromatogr. B Biomed. Sci. Appl..

[10] Xu M., Gao Z., Zhou Q., Lin Y., Lu M., Tang D. (2016). Terbium ion-coordinated carbon dots for fluorescent aptasensing of adenosine 5′-triphosphate with unmodified gold nanoparticles. Biosens. Bioelectron..

[11] Muthuraj B., Chowdhury S.R., Mukherjee S., Patra C.R., Iyer P.K. (2015). Aggregation deaggregation influenced selective and sensitive detection of Cu^2+^ and ATP by histidine functionalized water-soluble fluorescent perylene diimide under physiological conditions and in living cells. RSC Adv..

[12] Moschou E.A., Sharma B.V., Deo S.K., Daunert S. (2004). Fluorescence glucose detection: Advances toward the ideal in vivo biosensor. J. Fluoresc..

[13] Qu F., Guo X., Liu D., Chen G., You J. (2016). Dual-emission carbon nanodots as a ratiometric nanosensor for the detection of glucose and glucose oxidase. Sens. Actuators B.

[14] Liu S., Sun Y. (2007). Co-immobilization of glucose oxidase and hexokinase on silicate hybrid sol–gel membrane for glucose and ATP detections. Biosens. Bioelectron..

[15] Shan Z., Lu M., Wang L., MacDonald B., MacInnis J., Mkandawire M., Zhang X., Oakes K.D. (2016). Chloride accelerated Fenton chemistry for the ultrasensitive and selective colorimetric detection of copper. Chem. Commun..

[16] Cappella P., Giansanti V., Pulici M., Gasparri F. (2013). From “Click” to “Fenton” chemistry for 5-bromo-2′-deoxyuridine determination. Cytom. Part A.

[17] Stohs S.J., Bagchi D. (1995). Oxidative mechanisms in the toxicity of metal ions. Free Radic. Biol. Med..

[18] Wang H., Zhang H., Chen Y., Huang K., Liu Y. (2015). A label-free and ultrasensitive fluorescent sensor for dopamine detection based on double-stranded DNA templated copper nanoparticles. Sens. Actuators B.

[19] Shen P., Xia Y. (2014). Synthesis-modification integration: One-step fabrication of boronic acid functionalized carbon dots for fluorescent blood sugar sensing. Anal. Chem..

[20] De B., Karak N. (2013). A green and facile approach for the synthesis of water soluble fluorescent carbon dots from banana juice. RSC Adv..

[21] Wang F., Xie Z., Zhang H., Liu C., Zhang Y. (2011). Highly luminescent organosilane-functionalized carbon dots. Adv. Funct. Mater..

[22] Purbia R., Paria S. (2016). A simple turn on fluorescent sensor for the selective detection of thiamine using coconut water derived luminescent carbon dots. Biosens. Bioelectron..

[23] Chen J., Li Y., Lv K., Zhong W., Wang H., Wu Z., Yi P., Jiang J. (2016). Cyclam-functionalized carbon dots sensor for sensitive and selective detection of copper (II) ion and sulfide anion in aqueous media and its imaging in live cells. Sens. Actuators B.

[24] Zu F., Yan F., Bai Z., Xu J., Wang Y., Huang Y., Zhou X. (2017). The quenching of the fluorescence of carbon dots: A review on mechanisms and applications. Microchim. Acta.

[25] Pham A.N., Xing G., Miller C.J., Waite T.D. (2013). Fenton-like copper redox chemistry revisited: Hydrogen peroxide and superoxide mediation of copper-catalyzed oxidant production. J. Catal..

[26] Moffett J.W., Zika R.G. (1987). Reaction kinetics of hydrogen peroxide with copper and iron in seawater. Environ. Sci. Technol..

[27] Millero F.J., Sharma V.K., Karn B. (1991). The rate of reduction of copper (II) with hydrogen peroxide in seawater. Mar. Chem..

[28] Gray R.D. (1969). Kinetics of oxidation of copper (I) by molecular oxygen in perchloric acid-acetonitrile solutions. J. Am. Chem. Soc..

[29] Wang J., Jiang Y., Zhou C., Fang X. (2005). Aptamer-based ATP assay using a luminescent light switching complex. Anal. Chem..

[30] Zhu Y., Hu X., Shi S., Gao R., Huang H., Zhu Y., Lv X., Yao T. (2016). Ultrasensitive and universal fluorescent aptasensor for the detection of biomolecules (ATP, adenosine and thrombin) based on DNA/Ag nanoclusters fluorescence light-up system. Biosens. Bioelectron..

[31] Wang J., Wang L., Liu X., Liang Z., Song S., Li W., Li G., Fan C. (2007). A Gold Nanoparticle-Based Aptamer Target Binding Readout for ATP Assay. Adv. Mater..

[32] Tedsana W., Tuntulani T., Ngeontae W. (2013). A highly selective turn-on ATP fluorescence sensor based on unmodified cysteamine capped CdS quantum dots. Anal. Chim. Acta.

[33] Chen Z., Li G., Zhang L., Jiang J., Li Z., Peng Z., Deng L. (2008). A new method for the detection of ATP using a quantum-dot-tagged aptamer. Anal. Bioanal. Chem..

[34] Zuo X., Song S., Zhang J., Pan D., Wang L., Fan C. (2007). A target-responsive electrochemical aptamer switch (TREAS) for reagentless detection of nanomolar ATP. J. Am. Chem. Soc..

[35] Li C., Numata M., Takeuchi M., Shinkai S. (2005). A sensitive colorimetric and fluorescent probe based on a polythiophene derivative for the detection of ATP. Angew. Chem. Int. Ed..

[36] Lin T., Zhong L., Guo L., Fu F., Chen G. (2014). Seeing diabetes: Visual detection of glucose based on the intrinsic peroxidase-like activity of MoS_2_ nanosheets. Nanoscale.

[37] Jiang Y., Zhao H., Lin Y., Zhu N., Ma Y., Mao L. (2010). Colorimetric detection of glucose in rat brain using gold nanoparticles. Angew. Chem..

[38] Liu Q., Chen P., Xu Z., Chen M., Ding Y., Yue K., Xu J. (2017). A facile strategy to prepare porphyrin functionalized ZnS nanoparticles and their peroxidase-like catalytic activity for colorimetric sensor of hydrogen peroxide and glucose. Sens. Actuators B.

[39] Ling Y., Zhang N., Qu F., Wen T., Gao Z., Li N., Luo H. (2014). Fluorescent detection of hydrogen peroxide and glucose with polyethyleneimine-templated Cu nanoclusters. Spectrochim. Acta Part A.

[40] Zhang W., Ma D., Du J. (2014). Prussian blue nanoparticles as peroxidase mimetics for sensitive colorimetric detection of hydrogen peroxide and glucose. Talanta.

[41] Liu S., Tian J., Wang L., Luo Y., Sun X. (2012). A general strategy for the production of photoluminescent carbon nitride dots from organic amines and their application as novel peroxidase-like catalysts for colorimetric detection of H_2_O_2_ and glucose. RSC Adv..

