# Supplementary material for: Simultaneous Detection of Adenosine Triphosphate and Glucose Based on the Cu-Fenton Reaction
Source: Sensors (Basel). 2018 Jul 4;18(7):2151. doi: 10.3390/s18072151 (PMC6069456; doi:10.3390/s18072151)
Supplement: Supplementary file 1 [file sensors-18-02151-s001.zip › sensors-315442-supplementary.doc]

**Supplementary Information for**

**Simultaneous detection of adenosine triphosphate and glucose based on Cu-Fenton reaction**

Fei Qu^a,b *^, Jingwen Li^a,b^, Wenli Han^c^, Lian Xia^a,b^, Jinmao You^a,b,d^

^a^ The Key Laboratory of Life-Organic Analysis, Qufu Normal University, Qufu 273165, Shandong, China

^b^ Key Laboratory of Pharmaceutical Intermediates and Analysis of Natural Medicine, Qufu Normal University, Qufu 273165, Shandong, China

^c^ Laboratory Animal Center, Chongqing Medical University, Chongqing, China

^d^ Northwest Institute of Plateau Biology, Chinese Academy of Sciences, Xining 810001, China

*Corresponding author at: Qufu Normal University, Key Laboratory of Pharmaceutical Intermediates and Analysis of Natural Medicine, Qufu, China.

Tel.: +86 537 4456305; Fax: +86 537 4456305.

E-mail addresses: qufei3323@163.com (F. Qu)

**Fig. S1** Excitation-dependent fluorescence spectra of CNDs.

**Fig. S2** Fluorescence spectra of CNDs upon addition of different concentrations of Cu^2+^ (a) in the presence of H_2_O_2_ (5 mM) and the corresponding linear ranges of Cu^2+^ (b).

**Fig. S3** Fluorescence spectra of CNDs upon addition of different concentrations of Cu^2+^ in the absence of H_2_O_2_.

**Fig. S4** Fluorescence spectra of CNDs upon addition of different concentrations of H_2_O_2_ (a) in the presence of Cu^2+^ (2 µM) and the corresponding linear ranges of H_2_O_2_ (b).

**Fig. S5** Photographs of Cu^2+^-H_2_O_2_-TMB system with different concentrations of Cu^2+^ under visible light. The concentrations of H_2_O_2_ and TMB were 500 mM and 5 mM, respectively. The concentrations of Cu^2+^ were 0, 1, 2, 4, 6, 8 and 10 μM from left to right.

**Fig. S6** Influence of pH values on CNDs-Cu^2+^-H_2_O_2_ system. The concentrations of CNDs, H_2_O_2_ and Cu^2+^ were 0.014 mg/mL, 5 mM and 600 nM respectively

**Fig. S7** Influence of the incubation time between Cu^2+^and ATP. The concentrations of CNDs, H_2_O_2_, Cu^2+^ and ATP were 0.014 mg/mL, 5 mM, 600 nM and 1 µM respectively.

**Fig. S8** Fluorescence spectra of CNDs in the presence of different concentrations of glucose (a) and the corresponding linear range from 0.1 mM to 1 mM (b). The concentrations of CNDs, Cu^2+^ and GOx were 0.014 mg/mL, 600 nM and 10 U/mL respectively.
